# Failure of artesunate-mefloquine combination therapy for uncomplicated *Plasmodium falciparum *malaria in southern Cambodia

**DOI:** 10.1186/1475-2875-8-10

**Published:** 2009-01-12

**Authors:** William O Rogers, Rithy Sem, Thong Tero, Pheaktra Chim, Pharath Lim, Sinuon Muth, Duong Socheat, Frédéric Ariey, Chansuda Wongsrichanalai

**Affiliations:** 1 Naval Medical Research Unit #2, Komp. P2P/PLP-LITBANGKES, Jl. Percetakan Negara No. 29, Jakarta 10560, Indonesia; 2 National Malaria Center, Phnom Penh, Cambodia; 3 Institut Pasteur du Cambodge, 5 Boulevard Monivong, BP 983, Phnom Penh, Cambodia; 4 Ministry of Health Referral Hospital, Chouk District, Kampot Province, Cambodia; 5 Office of Public Health, USAID Regional Development Mission – Asia 93/1 Wireless Road, Bangkok 10330, Thailand

## Abstract

**Background:**

Resistance to anti-malarial drugs hampers control efforts and increases the risk of morbidity and mortality from malaria. The efficacy of standard therapies for uncomplicated *Plasmodium falciparum *and *Plasmodium vivax *malaria was assessed in Chumkiri, Kampot Province, Cambodia.

**Methods:**

One hundred fifty-one subjects with uncomplicated falciparum malaria received directly observed therapy with 12 mg/kg artesunate (over three days) and 25 mg/kg mefloquine, up to a maximum dose of 600 mg artesunate/1,000 mg mefloquine. One hundred nine subjects with uncomplicated vivax malaria received a total of 25 mg/kg chloroquine, up to a maximum dose of 1,500 mg, over three days. Subjects were followed for 42 days or until recurrent parasitaemia was observed. For *P. falciparum *infected subjects, PCR genotyping of *msp1*, *msp2*, and *glurp *was used to distinguish treatment failures from new infections. Treatment failure rates at days 28 and 42 were analyzed using both per protocol and Kaplan-Meier survival analysis. Real Time PCR was used to measure the copy number of the *pfmdr1 *gene and standard 48-hour isotopic hypoxanthine incorporation assays were used to measure IC_50 _for anti-malarial drugs.

**Results:**

Among *P. falciparum *infected subjects, 47.0% were still parasitemic on day 2 and 11.3% on day 3. The PCR corrected treatment failure rates determined by survival analysis at 28 and 42 days were 13.1% and 18.8%, respectively. Treatment failure was associated with increased *pfmdr1 *copy number, higher initial parasitaemia, higher mefloquine IC_50_, and longer time to parasite clearance. One *P. falciparum *isolate, from a treatment failure, had markedly elevated IC_50 _for both mefloquine (130 nM) and artesunate (6.7 nM). Among *P. vivax *infected subjects, 42.1% suffered recurrent *P. vivax *parasitaemia. None acquired new *P. falciparum *infection.

**Conclusion:**

The results suggest that artesunate-mefloquine combination therapy is beginning to fail in southern Cambodia and that resistance is not confined to the provinces at the Thai-Cambodian border. It is unclear whether the treatment failures are due solely to mefloquine resistance or to artesunate resistance as well. The findings of delayed clearance times and elevated artesunate IC_50 _suggest that artesunate resistance may be emerging on a background of mefloquine resistance.

## Background

The spread of drug resistant *Plasmodium falciparum *has complicated efforts to control malaria, and can lead to unnecessary mortality if ineffective drugs remain the standard of care after drug-resistant strains become established [1,2]. In south-east Asia, resistance to multiple anti-malarial drugs, including chloroquine, sulphadoxine-pyrimethamine, quinine, and mefloquine is common [3]. In the face of this situation, many countries in the region have adopted artemisinin combination therapies, including artesunate-mefloquine and artemether-lumefantrine. In both Thailand and Cambodia, rising failure rates for mefloquine monotherapy led to introduction of artesunate-mefloquine combination therapy in 1995 and 2000 [4,5], respectively. The combination was more effective than mefloquine monotherapy. Introduction of artesunate in combination with mefloquine, a drug against which resistance is already common in the region [6] may not protect against the development and spread of artesunate resistance. Indeed, preliminary reports from both sides of the Thai-Cambodian border have suggested that resistance to the artesunate-mefloquine combination may be emerging. In 2003, a regimen of 25 mg/kg mefloquine in two divided doses on day 0, and 12 mg/kg oral artesunate divided into two doses on day 0 and day 1, in Trat, Thailand, along the Cambodian border, produced an adequate clinical and parasitological response (ACPR) at day 28 in only 78.6% of 44 patients with uncomplicated *P. falciparum *malaria; at three other sites in Thailand the same regimen had an efficacy of > 90% [4]. In 2002 in Pailin, a Cambodian province bordering Trat, a similar regimen produced ACPR at 28 days in 85.7% of subjects [5]; in this study almost one third of patients received less than 12 mg/kg artesunate and 20 mg/kg mefloquine, because blister pack doses were administered according to age rather than to weight. Nonetheless, the ACPR was > 90% at sites outside of Pailin where the same dosing scheme was used. In 2004, using dosing based on weight rather than age, the 42 day ACPR was 79.3% in Pailin [5]. Both the Thai and Cambodian studies used directly observed therapy and the Cambodian study also used PCR correction for re-infection [7]. These findings suggest that resistance to artesunate-mefloquine may be emerging at the Thai-Cambodian border.

Although the Thai-Cambodian border is frequently cited as an epicenter for the emergence of anti-malarial drug resistance, there is no reason to think that new anti-malarial drug resistance genotypes are constrained to arise there. Mefloquine resistance is common in many areas outside the Thai-Cambodian border region, and artemisinin has been used as monotherapy in the Greater Mekong Area for hundreds of years. Four sites in Cambodia were recently surveyed for the presence of *P. falciparum *strains containing amplifications of the *pfmdr1 *gene, associated with mefloquine resistance. The highest prevalence of amplifications was found in Chumkiri, in Southern Cambodia, rather than at the Thai border in Pailin (unpublished data). In order to ask whether the high background of *pfmdr1 *amplification might favor emergence of resistance to artesunate-mefloquine, an *in vivo *efficacy study of combined 12 mg/kg artesunate and 25 mg/kg in uncomplicated *P. falciparum *malaria using the recommended WHO protocol for *in vivo *anti-malarial efficacy studies was conducted. In accordance with then current Ministry of Health recommendations the maximum dose used was 600 mg artesunate, 1,000 mg mefloquine. The efficacy of 25 mg/kg chloroquine, maximum dose 1,500 mg, as monotherapy for uncomplicated *P. vivax *malaria was studied simultaneously.

## Methods

### Study site

The study was conducted between August 2006 and February 2008 in Chumkiri District, Kampot Province, in southern Cambodia. Chumkiri is located approximately 100 km SSW of Phnom Penh and 50 km west of the Vietnamese border, at 10.7° latitude N, 104.3° longitude E. Chumkiri town is located on a plain used for rice cultivation and surrounded by forested mountains. The climate is tropical with a rainy season extending from May-June to November. Malaria cases occur throughout the year, but most cases occur during the rainy season. *Plasmodium falciparum *and *Plasmodium vivax *are present in roughly equal proportion.

### Study subjects

Subjects who presented to the Chumkiri Health Center with uncomplicated mono-infection by *P. falciparum *or *P. vivax *were recruited. Inclusion criteria for *P. falciparum *infected subjects were 1) uncomplicated *P. falciparum *infection with parasitaemia between 1,000 and 100,000 parasites/mm^3 ^as determined by counting parasites per 200 white blood cells or per 1,000 red blood cells in Giemsa-stained thick or thin blood films and multiplying by standard estimates of white and red blood cell counts, 5,000 wbc/mm^3 ^and 4,000,000 rbc/mm^3^, 2) age ≥ 1 year, 3) axillary temperature ≥ 37.5°C or a history of fever in the past 24 hours, and 4) negative urine pregnancy test for women. Exclusion criteria were 1) any sign of severe or complicated malaria according to World Health Organization criteria [8] 2) unwillingness to remain at clinic for directly observed therapy or to return for follow-up visits, 3) mixed species *Plasmodium *infection, and 4) breastfeeding. Inclusion and exclusion criteria for uncomplicated *P. vivax *infection were identical except that the lower limit of parasitaemia was 500 parasites/mm^3^.

### Anti-malarial therapy and follow-up

Subjects with uncomplicated falciparum malaria received a total dose of 12 mg/kg artesunate (MissionPharma, Lynge, Denmark, 50 mg tablets) in three daily doses and a total dose of 25 mg/kg mefloquine (PharmaDanica, Lynge, Denmark 250 mg tablets) in two doses eight hours apart, up to a maximum dose of 600 mg artesunate and 1,000 mg mefloquine. Subjects with uncomplicated vivax malaria received a total dose of 25 mg/kg chloroquine base (Nanjing Baijingyu Pharmaceutical Company, Ltd, Nanjing, China, 150 mg tablets) given 10 mg/kg on day 0 and day 1, and 5 mg/kg on day 2, up to a maximum total dose of 1,500 mg. All doses were directly observed. If the patient vomited within one hour of receiving a dose, the dose was repeated. The study physician evaluated each subject and collected blood for malaria thick and thin film examination on days 0,1,2,3,7,14,21,28,35, and 42, and at any time when the subject reported to the clinic with fever or other symptoms suggestive of malaria. Two microscopists read all blood smears; a third reference microscopist resolved any discrepancies. Patients with recurrent *P. falciparum *parasitaemias received oral quinine sulfate, 10 mg salt/kg three times daily, and tetracycline 1 g per day. Patients with new or recurrent *P. vivax *parasitaemia during the follow-up period received chloroquine as above.

### Parasite genotyping

For initial infections and recurrences with *P. falciparum*, DNA isolation and characterization of allelic polymorphisms in the genes encoding MSP1, MSP2, and GLURP by PCR were performed as described [9]. If any of the alleles from any of the three genes present in the sample from the day of recurrence were not present in the Day 0 sample, then the recurrence of parasitaemia was ascribed to a new infection rather than to treatment failure. The copy number of the *pfmdr1 *gene was determined by Real Time PCR (RT-PCR) as previously described [10]. In brief, a multiplexed assay using primers and a FAM-TAMRA (6-carboxyfluorescein 6-carboxy-tetra-methylrhodamine) labeled probe specific for *pfmdr1 *[11] and primers and a VIC-TAMRA (proprietary formula, ABI, Foster City, CA) probe [12] specific for β-tubulin was carried out, so that both genes could be assayed in the same reaction. The probes were obtained from MWG Biotech (High Point, NC) and the primers from Applied Biosystems (ABI, Foster City, CA). PCR reactions were performed on the Rotogene 6000 thermocycler (Corbett Life Science, New South Wales, Australia). Each plate included a sample from the control *P. falciparum *strains 3D7 and Dd2, which are known to have one and four *pfmdr1 *copies, respectively [13,14]. *Pfmdr1 *copy number was calculated as follows: copy number = (E_β-tubulin_)^CT(β-tubulin)^/(E_pfmdr1_)^CT(pfmdr1)^, where E and CT represent the efficiency and cycle threshold of the respective targets. It was assumed that E_β-tubulin _= 2; E_pfmdr1 _was then calculated using the CT_pfmdr1 _and CT_β-tubulin _values obtained with the 3D7 control. The results of the assay were accepted if the Dd2 control had a calculated *pfmdr1 *copy number between 3.5 and 4.5.

### In vitro drug resistance assay

The in vitro drug sensitivity of the *P. falciparum *isolates was assessed by use of a classical isotopic 48-hour test [15]. In brief, mefloquine and artesunate were obtained from IMTSSA (Institut de Medicine Tropical, Service de Santé des Armées, Marseille, France). Stock solutions of mefloquine and artesunate were prepared in methanol and further two-fold serial dilutions in distilled water (Biosedra, France). The final concentrations ranged from 0.05 to 51.2 nM for artesunate and 1 to 1024 nM for mefloquine. Two wells of a Falcon 96-well, flat-bottom plate (ATGC, France) were coated with each drug concentration, the plates were dried in a sterile cabinet, and were stored at 4°C until use. Their suitability for in vitro testing was monitored using reference strains of *P. falciparum *with known drug sensitivities. Fresh blood samples were washed three times with RPMI 1640 medium (GibcoTM, Invitrogen Corporation, France) by centrifugation (800 g, 10 min, 4°C) and then tested directly without culture adaptation. The infected erythrocytes (1.5% haematocrit, 0.1% – 1% parasitaemia) were suspended in complete RPMI medium supplemented with 10% of decomplemented human AB+ serum (Biomedia, France), buffered with 25 mM/l Hepes, 11 mM/l D-(+)-glucose 25 mM/l NaHCO3, and containing [8-^3^H] hypoxanthine (0.5 μci/well; Amersham Biosciences, France), and the mixture (200 μl per well) was distributed into the 96-well test plates pre-coated with anti-malarial drugs. Each plate included two drug-free control wells and one control well without parasites. The plates were incubated for 48 h at 37°C in a 5% CO_2 _atmosphere and the cells then lysed by freeze-thawing. After collection on glass-fiber filter paper using a cell harvester, the amount of [^3^H] hypoxanthine incorporated into the parasites' nucleoprotein was determined using a Wallac MicroBeta Trilux counter (Perkin Elmer, France). A log probit approximation was used to determine the 50% inhibitory concentration (IC_50_), defined as the concentration at which 50% of the incorporation of [^3^H] hypoxanthine was inhibited, as compared with the drug-free control wells.

### Statistical analysis

The primary outcome was recurrence of *P. falciparum *or *P. vivax *parasitaemia. Subjects who met World Health Organization criteria for early treatment failure (parasitaemia on day 2 > on day 0, parasitaemia on day 3 > 25% of parasitaemia on day 0, or fever and any parasitaemia on day 3) [7] but who cleared their parasitaemia by day 7 were not given additional treatment and were not analyzed as treatment failures unless they subsequently suffered recurrent parasitaemia. In the case of subjects with *P. falciparum *malaria, the results of both per protocol and Kaplan-Meier analysis are reported. In the per protocol analysis the proportion of treatment failures was calculated by dividing the total number of subjects with recurrent parasitaemias (with or without PCR correction for re-infection) by the number of subjects who either suffered recurrent parasitaemia or completed the full 42 day follow-up period. In the Kaplan-Meier analysis, subjects were censored from the point at which they 1) were lost to follow-up or 2) acquired a *P. vivax *infection. The proportion of treatment failures for both day 28 and day 42 is reported. In the case of subjects with uncomplicated *P. vivax *malaria the analysis was identical, except that no attempt was made to distinguish between recrudescence, re-infection, and relapse from the liver. Subjects were considered to have cleared parasitaemia if there were at least two sequential negative smears. The day on which the first such negative smear was observed was defined as the day of clearance. Because smears were not taken on days 4–6, subjects with a reported clearance day 7 may actually have cleared their parasitaemia on any day between day 4 and 7. All statistical analysis was performed with StataSE 8.0.

## Results

### In vivo efficacy

**Figure 1:**
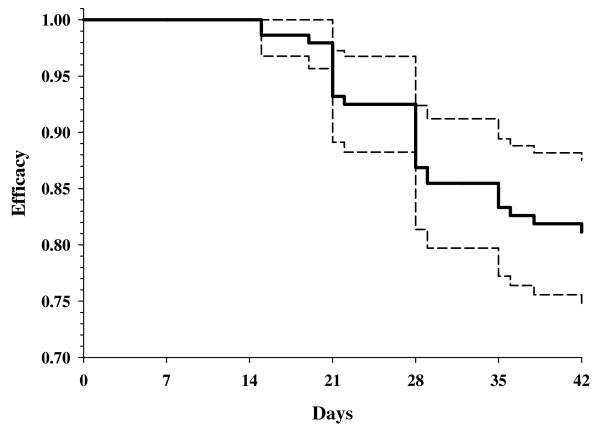
**Mefloquine-artesunate survival curve**. Shown is the proportion of subjects remaining free of *P. falciparum *following treatment with mefloquine artesunate. The point estimate (bold line) and 95% confidence intervals (dotted lines) were calculated by Kaplan-Meier survival analysis. Only PCR-confirmed treatment failures are included.

The baseline characteristics of the study subjects are shown in Table 1. Between August 2006 and December 2007, 151 subjects with uncomplicated *P. falciparum *malaria were enrolled. One subject withdrew before completing treatment. Of the 150 who completed treatment, 127 (84.7%) still had *P. falciparum *parasitaemia on day 1, 71 (52.7%) on day 2, and 17 (11.3%) on day 3. All subjects were free of parasitaemia by day 7. Of these 150 subjects, seven (4.7%) were lost to follow-up before completing 42 days follow-up. Among the remaining 143 subjects, 31 (20.7%) developed recurrent *P. falciparum *parasitaemia. None of the recurrent parasitaemias occurred before day 14. Based on PCR genotyping for MSP1, MSP2, and GLURP, 4 of these parasitaemias were judged to be new infections and 27 to be recrudescences. Of the 27 recrudescences, 24 were accompanied by fever (axillary temperature ≥ 37.5°C) and were classified as late clinical failures; the remaining 3 were classified as late parasitological failures. Four subjects met criteria for early treatment failure[7], but cleared their parasitaemia by day 7 without additional treatment. Of these four, three suffered recrudescences (two on day 21, one on day 28); one remained aparasitemic from day 7 through the end of the 42 day follow-up and was not counted as a treatment failure. Eight subjects (5.3%) developed *P. vivax *parasitaemia, one on day 32 and seven on day 42. Figure 1 shows the Kaplan-Meier survival curve for PCR-corrected artesunate-mefloquine efficacy, throughout the follow-up period. Table 2 shows failure rates at days 28 and 42, calculated on both a per protocol and survival analysis basis, with and without PCR correction for re-infection.

**Table 1:** Study subject demographics and parasitaemia

Species	Sex, male (%)	Age, yrs (S.D.)	Geometric Mean Parasitaemia, parasites/mm^3 ^(5, 95 centiles)
*P. falciparum*	141/151 (93.4%)	27.1 (9.5)	5857 (1250, 43375)

*P. vivax*	97/109 (89.0%)	21.2 (6.7)	5427 (1000, 11000)

**Table 2:** Failure rates for uncomplicated falciparum malaria treated with mefloquine artesunate

Analysis	PCR Correction	Failure Rate (%) Day 28 (95% CI)	Failure Rate (%) Day 42 (95% CI)
Kaplan-Meier	Yes	13.1 (8.5, 19.7)	18.8 (13.3, 26.2)
	
	No	15.1 (10.2, 22.0)	21.4 (15.5, 29.0)

Per Protocol	Yes	13.4 (7.8, 19.0)	19.4 (12.8, 26.0)
	
	No	15.2 (9.4, 21.0)	21.7 (14.9, 28.4)

One hundred ten subjects with uncomplicated vivax malaria were enrolled. One subject was withdrawn from the study on day 1 when he was found to have a mixed *P. vivax/P. falciparum *infection. Of the remaining 109 subjects, 79 (72.5%) still had *P. vivax *parasitaemia on day 1, 5 (4.6%) on day 2, and 1 (0.9%) on day 3. All subjects were free of parasitaemia by day 7. Two subjects were lost to follow-up, at 28 and 25 days. Among the 107 subjects who completed follow-up, 45 (42.1%) suffered recurrent *P. vivax *parasitaemia; all recurrent parasitaemias occurred between day 28 and day 42. No attempt was made to distinguish between recrudescence, relapse from the liver, and re-infection. None of the *P. vivax *subjects acquired *P. falciparum *infection during follow-up.

### Predictors of treatment failure

**Figure 2:**
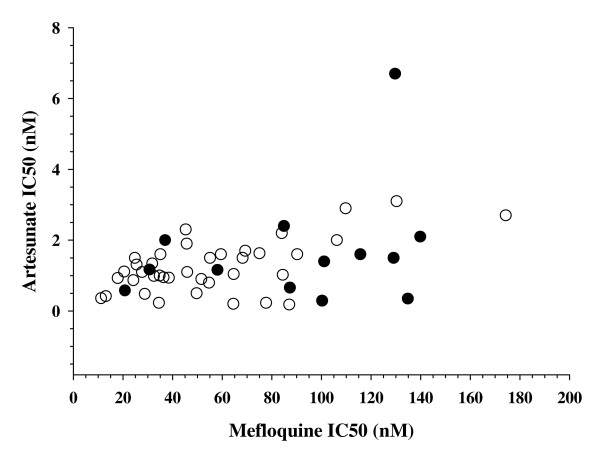
**In vitro susceptibilities to mefloquine and artesunate**. Shown are the IC_50 _for mefloquine and artesunate for parasite samples from patients who did (black circles) or did not (white circles) suffer a PCR-confirmed treatment failure.

For logistical reasons, it was possible to measure the IC_50 _for mefloquine and artesunate in samples from only 51 *P. falciparum *subjects, 38 subjects who did not suffer a recrudescence and 13 who did. Figure 2 shows the IC_50 _for mefloquine and artesunate for each of these samples. The mean mefloquine IC_50 _in subjects who suffered a recrudescence was 34 nM (95% CI 10–57) higher than in those who did not, 90 nM (95% CI 65–115 nM) versus 56 nM (95% CI 45–67 nM). The mean artesunate IC_50 _was also higher in subjects who suffered recrudescence, 1.7 nM (95% CI 0.7–2.7 nM), than in those who did not, 1.2 nM (95%CI 1.0–1.5 nM), but the difference was not statistically significant.

Amplification of the *pfmdr1 *gene has been associated with mefloquine resistance [16]. Table 3 shows the mean *pfmdr1 *copy number and the proportion of samples with estimated copy number > 1.5 in the samples from day 0 and from the day of recrudescence. Samples from the day 0 parasitaemia in subjects who ultimately suffered a recrudescence had a higher mean *pfmdr1 *copy number than those from subjects who did not (2.7 versus 1.9, P = 0.003, t test). The relative risk for recrudescence in subjects with *pfmdr1 *copy number > 1.5 was 3.5 (95% CI 1.4–8.8). Recrudescent samples had a higher mean copy number than the corresponding day 0 samples (3.5 versus 2.6, P = 0.0013, paired t test).

**Table 3:** Relationship between *pfmdr1 *copy number and risk of recurrence

Recrudescence	Number	*pfmdr*1 Copy # (95% CI) Day 0	Proportion with Copy # > 1.5 Day 0	*pfmdr*1 Copy # (95% CI) Day Recur	Proportion with Copy # > 1.5 Day Recur
No	122	1.9 (1.7–2.2)	0.45	3.0* (0.9–5.2)	0.75*

Yes	27	2.7 (2.1–3.2)	0.74	3.6 (2.9–4.3)	0.85

*Results from the 4 subjects with recurrent parasitaemia classified as re-infection by PCR

Table 4 shows the relationship between the day of clearance, the risk of recrudescence, amplification of the *pfmdr1 *gene, and the initial parasite density. Longer time to parasite clearance was associated with greater risk of subsequent recrudescence, a greater proportion of *pfmdr1 *amplification, and higher initial parasitaemia. Table 5 shows the results of multiple logistic regression analysis of factors associated with risk of recrudescence; higher initial parasitaemia, longer clearance time, and *pfmdr1 *copy number were independently associated with higher risk of recrudescence.

**Table 4:** Parasite clearance times

Day of Clearance*	Number (%) (95% CI)	Treatment Failure Proportion (95% CI)	*pfmdr1 *Copy # > 1.5 Proportion (95% CI)	Day 0 Parasite Density (parasites/mm^3^) Geometric Mean (95% CI)
1	23 (15.3) (9.5–21.2)	0.0 (0.0–0.13)	0.22 (0.05–0.39)	3099 (2049–4687)

2	56 (37.3) (29.5–45.1)	0.16 (0.06–0.26)	0.52 (0.39–0.65)	5336 (4089–6964)

3	54 (36.0) (28.2–43.8)	0.19 (0.09–0.29)	0.57 (0.44–0.70)	7741 (5887–10179)

7	17 (11.3) (6.2–16.5)	0.47 (0.23–0.72)	0.59 (0.36–0.82)	8061 (4912–13230)

*Parasitaemia was considered to have cleared after two negative smears. The day on which the first of these two negative smears was observed was counted as the day of clearance.

**Table 5:** Multiple logistic regression model of risk factors for recrudescence.

Risk factor	Odds ratio* (95% CI)	P value
Log parasite density	3.40 (1.12, 10.3)	0.031

Day of clearance	1.48 (1.13, 1.94)	0.004

*Pfmdr1 *copy number	1.92 (1.32, 2.79)	0.001

*This represents the increment in the odds ratio for recrudescence for each single unit increase in the risk factor, one log of parasitaemia density, one additional day for clearance, or one additional copy of *pfmdr1*.

## Discussion

An unexpectedly high failure rate for artesunate-mefloquine treatment of uncomplicated falciparum malaria was found at a site in Cambodia far from the Thai-Cambodian border, frequently considered a hot spot for the emergence of anti-malarial drug resistance. The failure rates at 28 and 42 days, calculated using Kaplan-Meier survival analysis and PCR correction for re-infection, were 13.1% (95% CI 8.5–19.7%) and 18.8% (95% CI 13.3–26.2%), respectively. Although it was not possible to measure circulating drug levels during therapy, all doses were directly observed, and the drugs were obtained through the Cambodian Ministry of Health from suppliers following current Good Manufacturing Practices. Thus, while it is formally possible that the unexpectedly high failure rates are due to adulterated, defective, or counterfeit anti-malarials, it seems unlikely. The study used the then current maximum dose of mefloquine, 1,000 mg. It is possible that efficacy would have been higher had we used a maximum of 1,250 or 1,500 mg. If treatment failures were related to a low dose/weight of mefloquine, it would have been expected that heavier subjects would be at greater risk of failure. The mean weight, however, of subjects who had an ACPR (50.6 kg; 95% CI 49.0, 52.3), was not significantly different from those who did not (51.0 kg; 95% CI 47.8, 54.2); similarly the mean administered mefloquine dose per kg did not differ significantly between ACPR and failure (19.8 mg/kg vs. 19.7 mg/kg, P = 0.85, t-test). The risk of failure was the same in subjects < 40 kg, 40–50 kg, or > 50 kg, and in regression analysis there was no relationship between either weight or actual mefloquine dose per kg and time to clearance or risk of recrudescence. It therefore seems unlikely that increasing the maximum mefloquine dose would have significantly changed the observed failure rates.

It is possible that PCR correction in fact led to an underestimation of the failure rate. Malaria transmission is not intense at this study site; most infections (> 80%) contained only a single genotype (data not shown). None of the 109 subjects enrolled with uncomplicated *P. vivax *malaria and treated with chloroquine acquired new *P. falciparum *infection during the 42 day follow-up period, suggesting that the *P. falciparum *attack rate is relatively low. PCR genotyping may miss minor parasite populations present on day 0 which may be selected during treatment, thus incorrectly characterizing treatment failures as new infections. That such selection occurred is suggested by the observation (Table 3) that the mean *pfmdr1 *copy number increased from day 0 to the day of recurrence, even in the 4 cases classified by PCR as re-infection. If these four cases in fact represent additional treatment failures, then the 28 and 42 day failure rates would be 15.1% (95%CI 10.2–22.0%) and 21.4% (95% CI 15.5–29.0%), respectively.

Use of non-study anti-malarial drugs during follow-up might also lower the measured treatment failure rate. Although subjects were instructed not to take anti-malarial drugs during the follow-up period, except those provided as treatment for documented recurrences by the study team, and although none reported having done so, we cannot exclude the possibility that some subjects may have taken non-study anti-malarial drugs. Such unrecorded treatment might have reduced the apparent treatment failure rate. In that case the failure rates reported herein would be an underestimate.

It is not clear whether the resistance observed here is due entirely to resistance to mefloquine, or whether there is also a contribution of resistance to artesunate. It is well known that a 3 day regimen of artesunate monotherapy for *P. falciparum *has a high risk of late recrudescence [17,18]. It is possible that mefloquine resistance is effectively converting the artesunate-mefloquine combination therapy used here into artesunate monotherapy. Indeed, both the presence of amplification of the *pfmdr1 *gene and elevated mefloquine IC_50 _were predictive of treatment failure. It may be that all the observed treatment failures are due solely to mefloquine resistance. There was, however, some evidence suggestive of resistance to artesunate. First, pure mefloquine resistance should not greatly affect the time to parasite clearance in subjects treated with artesunate-mefloquine combination therapy. Trials of artesunate monotherapy [19,20] and artesunate-mefloquine combination therapy [21] have generally found more rapid parasite clearance than found at this site. For example, in a study conducted in 2001 on the western border of Thailand [21], treatment with the same artesunate-mefloquine regimen used here produced parasite clearance by day 2 in 98.4% of uncomplicated *P. falciparum *infections. Similarly, in a study carried out in northern Lao in 2003 [22], the same artesunate-mefloquine regimen cleared 98.1% of subjects by day 2. Although clearance times with artesunate-mefloquine may be slightly faster than with artesunate monotherapy [19], even artesunate monotherapy is expected to clear parasitaemia in > 90% of subjects by day 2 [19,20]. In contrast, only 53% of subjects in the current study treated with artesunate-mefloquine were free of parasitaemia by day 2. Second, although the artesunate IC_50 _values were not strongly predictive of treatment failure, one sample with markedly elevated IC_50 _for both artesunate (6.7 nM) and mefloquine (130 nM) was identified. It thus appears possible that, at this study site, artesunate resistance is beginning to emerge on a background of pre-existing mefloquine resistance. If recent reports of declining efficacy of artesunate-mefloquine in Trat, Thailand [4], and Pailin, Cambodia [5], near the Thai-Cambodian border similarly reflect early emergence of artesunate resistance, then that resistance may not be confined to a small focus on the Thai-Cambodian border. Regardless of the specific target or mechanism of resistance, the failure rates observed here for artesunate-mefloquine, if confirmed elsewhere, suggest that it may be necessary to use a different partner drug in artemisinin combination therapy in Cambodia.

Current Cambodian Ministry of Health treatment guidelines do not include primaquine treatment for vivax malaria because of the difficulty in screening for G6PD deficiency before treatment. It is not surprising that frequent recurrent *P. vivax *parasitaemia by day 42 (45/110, 41.3%) was found. All of the recurrent parasitaemias occurred after day 28 and all but 2 after day 35. Given the late timing of the recurrences, the absence of radical treatment to eliminate hypnozoites from the liver, and the lack of blood chloroquine levels on the day of recurrence, it is impossible to draw any conclusions about chloroquine resistance. It is clear, however, that subjects with vivax malaria treated with chloroquine alone are at substantial risk of recurrent morbidity; further studies including primaquine treatment and measurement of drug levels will be required to determine whether chloroquine resistance, reported elsewhere in south-east Asia, is a problem in Cambodia.

## Competing interests

The authors declare that they have no competing interests.

## Authors' contributions

WOR participated in study design and data collection, analyzed the data and drafted the manuscript; RS performed the *pfmdr1 *copy number assays and performed quality control on the data; TT examined and performed follow-up of study subjects after treatment; PC participated in the in vitro drug resistance assays; PL participated in the in vitro drug resistance assays; SM participated in study design; DS participated in study design; FA oversaw the in vitro drug resistance and *pfmdr1 *copy number assays and participated in data analysis; CW conceived the study, participated in study design, and performed quality control of data. All authors contributed to the critical review of the manuscript and agree to submission.
